# Off-State Performance Characterization of an AlGaN/GaN Device via Artificial Neural Networks

**DOI:** 10.3390/mi13050737

**Published:** 2022-05-05

**Authors:** Jing Chen, Yufeng Guo, Jun Zhang, Jianhua Liu, Qing Yao, Jiafei Yao, Maolin Zhang, Man Li

**Affiliations:** 1 College of Integrated Circuit Science and Engineering, Nanjing University of Posts and Telecommunications, Nanjing 210023, China; cjjcnjupt@163.com (J.C.); jhliu_njupt@163.com (J.L.); yqdeemail@163.com (Q.Y.); jfyao@njupt.edu.cn (J.Y.); zhangml5277@163.com (M.Z.); qiqing0206@njupt.edu.cn (M.L.); 2 National and Local Joint Engineering Laboratory for RF Integration and Micro-Packaging Technologies, Nanjing University of Posts and Telecommunications, Nanjing 210023, China

**Keywords:** artificial neural network, off-state I–V curve, breakdown performance, AlGaN/GaN HEMT

## Abstract

Due to the complexity of the 2D coupling effects in AlGaN/GaN HEMTs, the characterization of a device’s off-state performance remains the main obstacle to exploring the device’s breakdown characteristics. To predict the off-state performance of AlGaN/GaN HEMTs with efficiency and veracity, an artificial neural network-based methodology is proposed in this paper. Given the structure parameters, the off-state current–voltage (I–V) curve can therefore be obtained along with the essential performance index, such as breakdown voltage (BV) and saturation leakage current, without any physics domain requirement. The trained neural network is verified by the good agreement between predictions and simulated data. The proposed tool can achieve a low average error of the off-state I–V curve prediction (Ave. Error < 5%) and consumes less than 0.001‰ of average computing time than in TCAD simulation. Meanwhile, the convergence issue of TCAD simulation is avoided using the proposed method.

## 1. Introduction

AlGaN/GaN devices have gained successful application in high-power fields due to their better than silicon performance [1,2]. One of the most essential performance parameters for a GaN-based power device is its capability to handle a high voltage in its off state [3]. The success of the designing and exploration of Si-based power devices in the past few decades has mainly relied on the mature combination of technology computer-aided design (TCAD) tools and analytical models. This combined tool is currently not that applicable in the exploration of AlGaN/GaN HEMTs, who have much more complicated device physics. The simulations using commercial TCAD tools not only require the users for their abundant experience in physics and simulations. These time difference (TD) method- or Monte Carlo (MC) method-based simulations are also time-consuming and poor in convergence. Moreover, due to the complicated coupling effects resulting from the stacked structure and 2D distribution of charges, considering the influence of background carriers, traps, and interface states in AlGaN/GaN HEMT, such a defect of the conventional TCAD method is therefore amplified [4,5]. For the same reason, the physical analytical models which are expected to be capable of effectively characterizing the correlation between structure parameters and off-state performance are yet to be presented, demonstrating the difficulty of characterizing the off-state performance.

However, the machine learning (ML) techniques that have emerged in recent years provide a potential means to effectively predict these devices’ performance without using physical-based models [6,7,8]. By using an artificial neural network (ANN), the ML-based methods can explore the latent relationship between input and output data via training a neural network that is constructed by several hidden-layer. Once the neural network is trained adequately by using enough valid data, the output data can therefore be predicted correctly and efficiently [9,10]. In this case, one group of input data consists of a set of device structure parameters and bias conditions. Nowadays, ML-based methods are employed to predict one or several core parameters such as breakdown voltage (BV) and on-resistance (Ron) [11]. Yet, the answer to the more complicated question of how the structure parameters of an AlGaN/GaN HEMT affect the off-state I–V curve and breakdown performance remains unclear.

In this paper, an efficient numerical method using a multi-layer ANN framework is proposed to characterize the off-state performance of AlGaN/GaN HEMTs. The proposed method features its capability to predict the complicated off-state performance of AlGaN/GaN HEMTs swiftly. The effectiveness and veracity of the ANN-based numerical methodology are effectively verified by their good agreement with calibrated TCAD simulations. The average error of the off-state I–V curve between the predictions and simulations is less than 5%. Meanwhile, since no physical models are employed in the proposed numerical approach, the average computing time using the proposed method is only 10^−6^ of that using the TCAD tool.

## 2. Off State Performance Prediction

**Figure 1:**
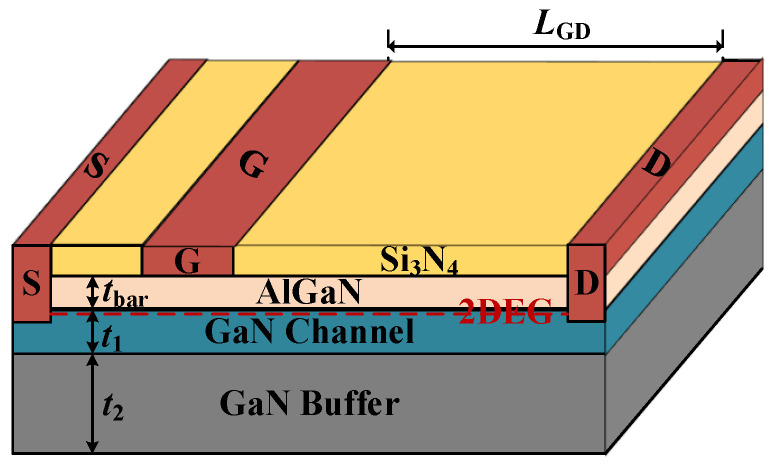
Schematic diagram of AlGaN/GaN HEMT structure.

As shown in Figure 1, an AlGaN/GaN HEMT is composed of a stacked structure of the AlGaN barrier layer, GaN channel layer, GaN buffer layer. Among them, a 2D layer of high-density polarization charge is formed at the AlGaN/GaN interface. This two-dimensional electron gas (2DEG) not only determines the on-state characteristic but also plays an important part in off-state behavior. Table 1 gives the basic material properties of GaN for the simulation. Meanwhile, unlike the conventional Si-based lateral power devices, the GaN-based power devices’ off-state breakdown performance is influenced by multiple 2D effects simultaneously. Such a collective 2D coupling effect is not only determined by the value of the devices’ structure parameters, but also the unusual/unclear 2D physical mechanism, thus resulting in a distinctive off-state characteristic of the AlGaN/GaN HEMT.

**Figure 2:**
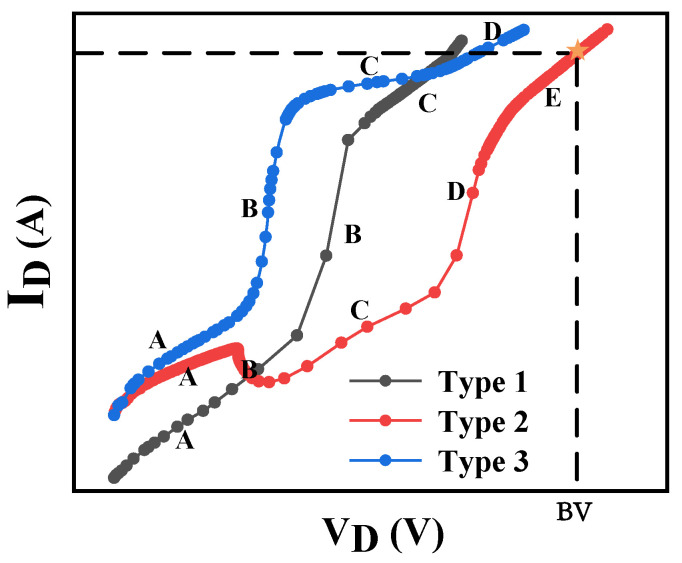
The off-state I–V curve of AlGaN/GaN HEMT.

In particular, as shown in Figure 2, the simulated off-state I–V curve indicates that the analysis of the off-state characteristics on the device is quite complicated. The three plots in Figure 2 are exported from the commercial TCAD tool Sentaurus. The breakdown can occur under various states, such as partially depleted breakdown and fully depleted breakdown. When the breakdown occurs, the depletion region may be at different depletion stages, and the current trend will also occur differently. In particular, the condition can be ascribed to three different types according to the changing trend of leakage current with drain voltage [12,13]. Especially for the case of type 2, as shown in Figure 2, there are five different stages in the trend of leakage current, which also demonstrate the superior difficulty of the off-state characteristics analysis. Yet, the prediction of the off-state performance is essential to a power device such as an AlGaN/GaN HEMT. Hence, we propose the ANN-based method to characterize off-state characteristics. The proposed approach is expected to provide an efficient and accurate prediction tool by using the data to explore the latent relationship between the device and its off-state performance without physical requirements.

**Figure 3:**
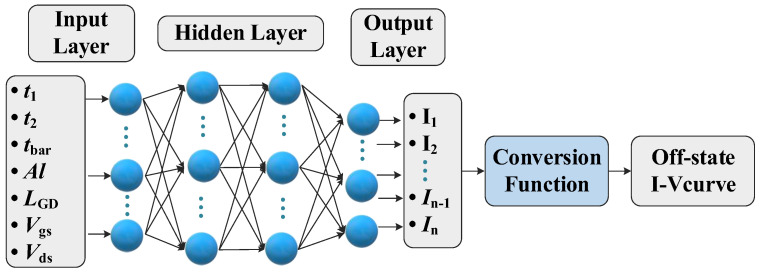
The overall flow of the proposed framework for off-state performance characterization.

For the ANN architecture used in this paper, the input neurons represent the applied voltage (i.e., *V*_gs_, *V*_ds_) and structure parameter (i.e., *t*_1_, *t*_2_, *t*_bar_ et al.). Figure 3 shows the overall flow of the proposed framework for off-state performance characterization. The number of hidden layers and the neurons are the training hyperparameters (i.e., weight, bias), which are subject to the tuning process to realize the optimal solution according to the complexity of the prediction task. The neurons of the output layer represent the current-related features, which are used as the input of the conversion function. As for the configurations for the proposed ANN, first, ReLU is employed as the nonlinear activation function [14]. Then, Adam optimizer is used to perform the error backpropagation process by updating the training parameters [15]. In addition, an adaptive learning rate is utilized to control the optimization process [16]. Moreover, the error between the output layer and the targets can be evaluated as the mean square error (*MSE*) [17], which can be formulated as:

(1) $$MSE=\frac{1}{N}\overset{N}{\underset{i=0}{\sum }}{\left(I_{i}-I_{i}^{t}\right)}^{2}$$

where *N* is the total training data number and *I_i_* and $I_{i}^{t}$ are the predicted feature and target value of the *i*th sample, respectively.

When the device is under the off-state condition, as the applied drain voltage *V*_d_ increases, the span of the current change will cover several orders of magnitude (i.e., from 10^−16^ to 10^−9^), which will hinder the fitting ability between the training dataset and the neural network. Thus, to optimize the fitting capability, a conversion function (CF) is introduced. Mathematically, the conversion function in this work can be expressed as:

(2) $$I_{\mathrm{ds}}=I_{0}\cdot 10^{-I_{i}}$$

where *I*_0_ denotes the standardization factor and *I_i_* represents the output features of the output layer. The components of the conversion function can effectively guarantee that the range of output features *I_i_* lie within a small range. It can not only eliminate the adverse effects caused by singular sample data, but also can accelerate the speed of gradient descent to find the optimal solution, which will improve the prediction accuracy. The dataset for the model training was generated by TCAD simulation. A total of 950 groups of devices were collected for off-state characteristics; 80% of the dataset was used to train the model, while the remaining part was used to evaluate the model. Table 2 shows the variation range of structural parameters. By combining the applied voltage (i.e., *V*_gs_, *V*_ds_) and structure parameters (i.e., *t*_1_, *t*_2_, *t*_bar_ et al.), the dataset for off-state performance characterization contains 78,595 samples. In addition, the ANN algorithm is established by using the available algorithms in the standard package Tensorflow and then utilized to characterize the off-state performance of AlGaN/GaN HEMT.

## 3. Results and Discussion

### 3.1. ANN Model Prediction for Off-State I–V Curve

**Figure 4:**
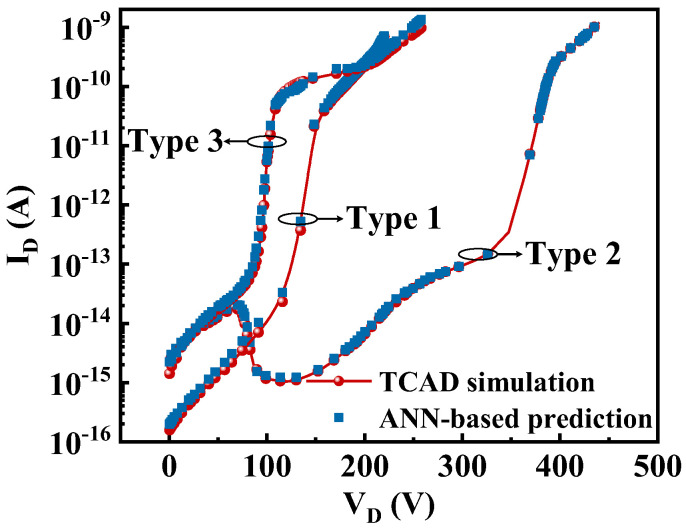
The predicted off-state I–V curve of the ANN-based framework.

Figure 4 shows the comparison between the predicted results from the ANN-based framework and targets simulated by TCAD simulation. As shown in Figure 4, clearly, with the increase in the drain voltage, the different structures will go through different states to achieve breakdown. The three different types of off-state I–V curves corresponding to Figure 2 are given. The red round point line represents the off-state performance obtained by TCAD simulation, and the blue square point results, representing the predictions of the proposed ANN-based method, are almost in good agreement with the target simulation results, demonstrating the capability of the proposed approach. Moreover, all of these I–V curves can be effectively characterized by the ANN-based framework without physics domain knowledge.

**Figure 5:**
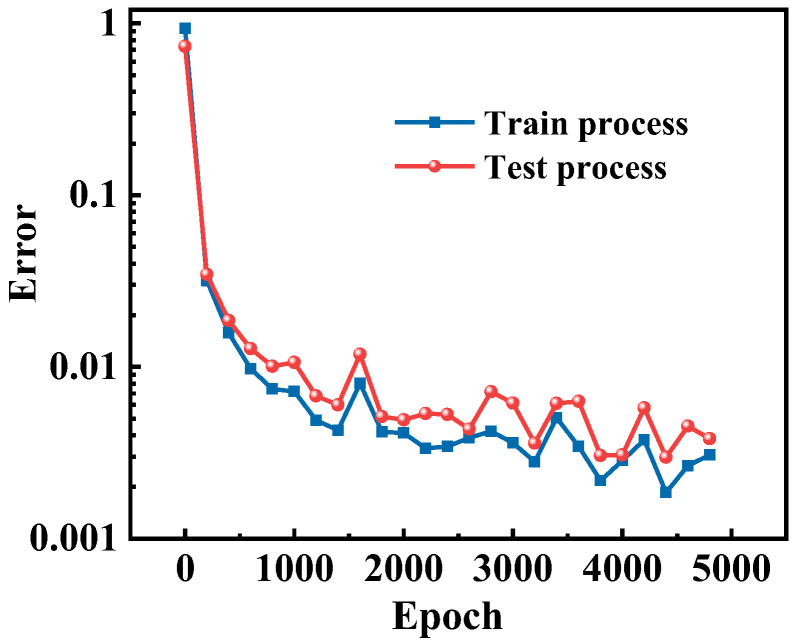
Off-state I–V curve prediction error changes corresponding to different training epochs.

Figure 5 shows the off-state I–V curve prediction error changes under different training epochs. In the initial stage of the training process, the prediction error is relatively large, so the error drastically changes to optimize the loss function by updating the training weights and bias. As the training epoch increases, the error changes tend to converge, validating the effectiveness of the model optimization process. In addition, since the ANN-based model optimization is a dynamic tuning process by updating the training parameters, it is reasonable to have small fluctuations accompanying the training process. Meanwhile, the similar changes in training and testing errors indicate that no overfitting occurs during the model generalization process.

### 3.2. The Effectiveness of Introducing Conversion Function

**Figure 6:**
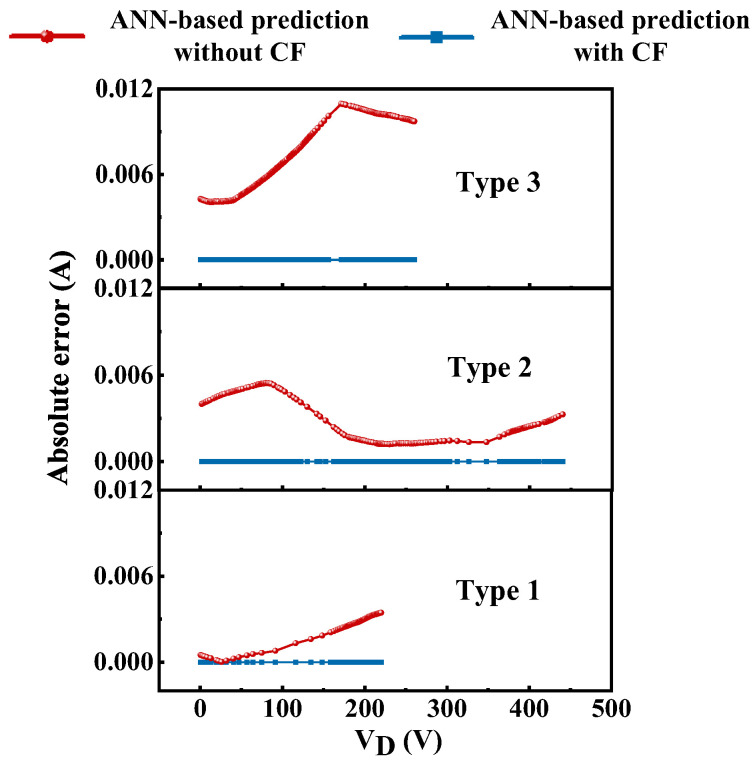
The predicted results with and without the conversion function (CF).

To examine the effectiveness of introducing the conversion function, we explore the prediction results without considering the conversion function (CF). Figure 6 gives the results by only using the ANN framework with and without the conversion function. The results represent the absolute error between the ANN-based predictions and TCAD simulation. The closer to zero, the better the results. As the figure shows, the absolute error between the predictions and targets of the prediction framework with CF under the three types of condition are close to zero. However, those results without CF fail to capture the relationship between the input features (i.e., *V*_ds_, *t*_1_, *t*_2_, *t*_bar_, etc.) and the drain current. Since the drain current covers a wide range of dimensional units, such a case will severely affect the convergence behavior of the ANN framework, thereby leading to training failure. However, by utilizing the conversion function to exponentially process the output results, the magnitude range of the data can be narrowed to a small range, making the network training more accurate and efficient.

**Figure 7:**
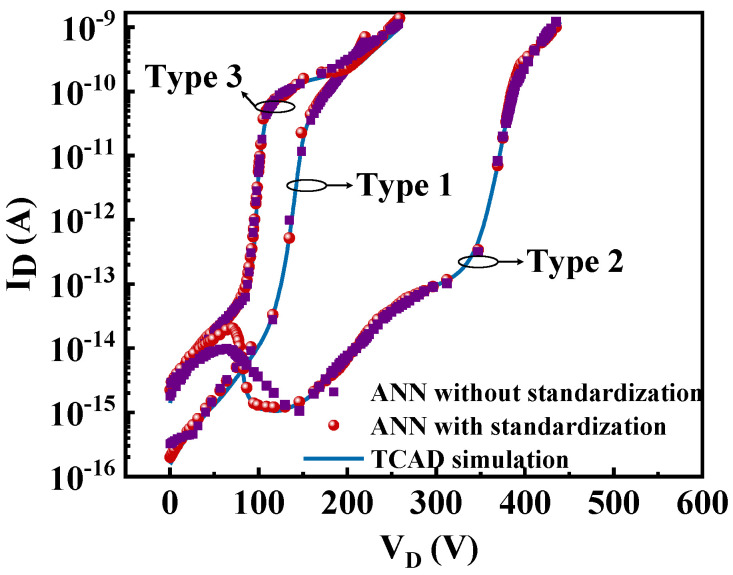
The predicted results consider the conversion function without the standardization factor.

Moreover, Figure 7 shows the results considering the conversion function without the standardization factor *I*_0_, as in Equation (1). The red circular points represent the results considering the standardization factor, while the purple square ones represent the result when the standardization factor is not considered. It can be observed that a better agreement between the targets and predictions can be achieved with the standardization factor. Obviously, especially for the case of type 2, compared to the results with the standardization factor, there is still a slight diversion from the target using the ANN-based prediction without the standardization factor. Thus, to further improve the prediction ability, the standardization factor *I*_0_ is introduced to narrow the range of the output features, thereby optimizing the model training process.

### 3.3. Breakdown Voltage Extraction

**Figure 8:**
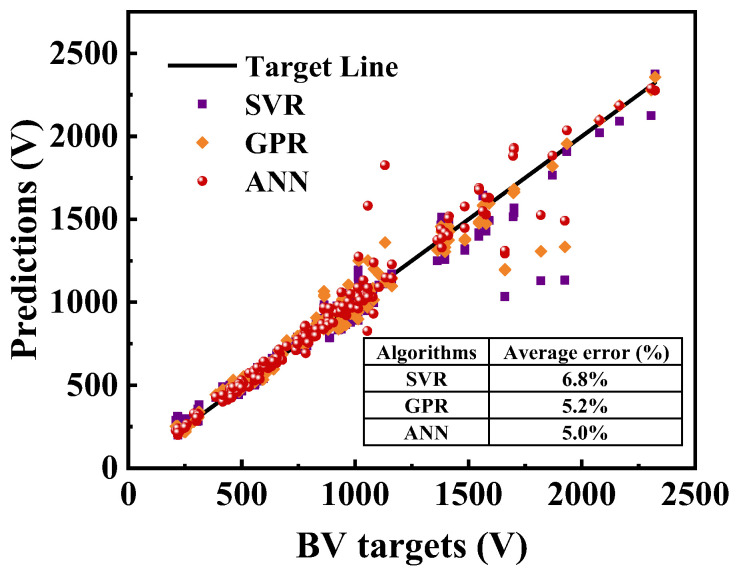
The breakdown voltage prediction results from the off-state I–V curve.

Figure 8 shows the breakdown voltage prediction results from the off-state I–V curve. The points represent the results obtained by the various machine learning algorithms, including support vector regression (SVR), Gaussian process regression (GPR), and the proposed ANN-based method. The solid black line represents the results where the targets are equal to the predictions. The closer the points are to the line, the higher the prediction accuracy of the results is. It is important to note that almost all predicted points representing breakdown voltage are located on the target line with these machine learning approaches. Yet, by means of the numerical calculation, the use of the proposed ANN method allows an average prediction error of around 5%, which shows superior capability for the prediction task.

### 3.4. Time Comparison

To exhibit the efficiency of the proposed method, the time taken to predict the off-state I–V curve is also analyzed. By using the simulation tool to obtain the off-state performance, the average time for the various collected test structures is about 8 h or even longer due to convergence issues. However, for all different device structures, the time to predict such performance remains at 0.01 s with the ANN-based method. The computing time speeds up by more than 10^6^ times compared to the TCAD tools. Moreover, the convergence issue which is associated with the TCAD tools is totally avoided by the proposed method.

## 4. Conclusions

In this paper, we propose an ANN-based predictive framework to predict the off-state curve of AlGaN/GaN HEMTs. By employing the proposed method, the complicated off-state performance can be accurately predicted without utilizing time-consuming TCAD simulation tools. The numerical results show that the proposed method is not only capable of tracing off-state I–V curves, but also appliable for predicting the critical parameter based on the structural parameters. Significantly, the average error of off-state I–V curve prediction is less than 5%. Moreover, since no physical equations and models are employed in the proposed approach, the convergence issue that easily occurs in TCAD tools is absolutely avoided. Meanwhile, by using the proposed method, the average computing time is only 10^−6^ of that using the TCAD tool.

## Figures and Tables

**Table 1:** The basic material properties of GaN.

Property	GaN
Bandage (eV)	3.4
Critical electric field (MV/cm)	3.3
Thermal conductivity (W/(cm·K))	1.3
Electron carrier mobility (cm^2^/(V·s))	2000
Electron saturation velocity (10^7^ cm/s)	2.5

**Table 2:** AlGaN/GaN HEMTs structural parameters and their variation ranges.

Structural Parameters	Range
GaN channel thickness *t*_1_, (μm)	[0.2, 0.8]
GaN buffer thickness *t*_2_, (μm)	[0.6, 5.4]
AlGaN barrier thickness *t*_bar_, (μm)	[0.005, 0.02]
The length of Gate to Drain, *L*_GD_ (μm)	[5, 25]
Al composition, *Al*	[0.15, 0.25]

## Data Availability

Not applicable.

## References

[1] Zhang S., Liu X., Wei K., Huang S., Chen X., Zhang Y., Zheng Y., Liu G., Yuan T., Wang X. (2021). Suppression of Gate Leakage Current in *Ka*-Band AlGaN/GaN HEMT with 5-Nm SiN Gate Dielectric Grown by Plasma-Enhanced ALD. IEEE Trans. Electron Devices.

[2] Green B.M., Chu K.K., Chumbes E.M., Smart J.A., Shealy J.R., Eastman L.F. (2000). The Effect of Surface Passivation on the Microwave Characteristics of Undoped AlGaN/GaN HEMTs. IEEE Electron Device Lett..

[3] del Alamo J.A., Lee E.S. (2019). Stability and Reliability of Lateral GaN Power Field-Effect Transistors. IEEE Trans. Electron Devices.

[4] Vigneshwara Raja P., Nallatamby J.-C., DasGupta N., DasGupta A. (2021). Trapping Effects on AlGaN/GaN HEMT Characteristics. Solid-State Electron..

[5] Soni A., Shikha S., Shrivastava M. (2019). On the Role of Interface States in AlGaN/GaN Schottky Recessed Diodes: Physical Insights, Performance Tradeoff, and Engineering Guidelines. IEEE Trans. Electron Devices.

[6] Yang Q., Qi G., Gan W., Wu Z., Yin H., Chen T., Hu G., Wan J., Yu S., Lu Y. (2021). Transistor Compact Model Based on Multigradient Neural Network and Its Application in SPICE Circuit Simulations for Gate-All-Around Si Cold Source FETs. IEEE Trans. Electron Devices.

[7] Dhillon H., Mehta K., Xiao M., Wang B., Zhang Y., Wong H.Y. (2021). TCAD-Augmented Machine Learning with and without Domain Expertise. IEEE Trans. Electron Devices.

[8] Mehta K., Wong H.-Y. (2021). Prediction of FinFET Current-Voltage and Capacitance-Voltage Curves Using Machine Learning with Autoencoder. IEEE Electron Device Lett..

[9] Wang Z., Li L., Yao Y. (2021). A Machine Learning-Assisted Model for GaN Ohmic Contacts Regarding the Fabrication Processes. IEEE Trans. Electron Devices.

[10] Wang J., Kim Y.-H., Ryu J., Jeong C., Choi W., Kim D. (2021). Artificial Neural Network-Based Compact Modeling Methodology for Advanced Transistors. IEEE Trans. Electron Devices.

[11] Chen J., Alawieh M.B., Lin Y., Zhang M., Zhang J., Guo Y., Pan D.Z. (2020). Powernet: SOI Lateral Power Device Breakdown Prediction with Deep Neural Networks. IEEE Access.

[12] Yu X., Ni J., Li Z., Zhou J., Kong C. (2014). Reduction in Leakage Current in AlGaN/GaN HEMT with Three Al-Containing Step-Graded AlGaN Buffer Layers on Silicon. Jpn. J. Appl. Phys..

[13] Wang C., He Y.-L., Ding N., Zheng X.-F., Zhang P., Ma X.-H., Zhang J.-C., Hao Y. (2014). Simulation and Experimentation for Low Density Drain AlGaN/GaN HEMT. Chin. Phys. Lett..

[14] Schmidt-Hieber J. (2020). Nonparametric Regression Using Deep Neural Networks with ReLU Activation Function. Ann. Stat..

[15] Zhang Z. (2018). Improved Adam Optimizer for Deep Neural Networks. Proceedings of the 2018 IEEE/ACM 26th International Symposium on Quality of Service (IWQoS).

[16] Takase T., Oyama S., Kurihara M. (2018). Effective Neural Network Training with Adaptive Learning Rate Based on Training Loss. Neural Netw..

[17] Alejo R., García V., Pacheco-Sánchez J.H. (2015). An Efficient Over-Sampling Approach Based on Mean Square Error Back-Propagation for Dealing with the Multi-Class Imbalance Problem. Neural Process. Lett..

